# 775. Risk Factors for Healthcare Associated Central Line-Associated Bloodstream Infection (CLABSI) to Identify Novel Infection Prevention Areas - A Case-Control Study

**DOI:** 10.1093/ofid/ofab466.972

**Published:** 2021-12-04

**Authors:** Aung Myat Oo, Pin Hong Jin, Edwin Philip, Molly Kue Bien How, May Kyawt Aung, Lai Chee Lee, Shalvi Arora, Indumathi venkatachalam, Jean Xiang Ying Sim, Moi Lin Ling, Yong Yang

**Affiliations:** Singapore General Hospital, Singapore

## Abstract

**Background:**

The International Nosocomial Infection Control Consortium surveillance reported Central line-associated bloodstream infection (CLABSI) rate of 4.1 per 1000 central-line days in 703 ICUs in 50 countries.

**Methods:**

At the Singapore General Hospital (SGH) a 1,700-bed tertiary care hospital, we conducted a retrospective matched case control study over a 3-year period from 2018 to 2020, to identify risk-factors associated with the development of healthcare associated CLABSI in adult inpatients. Cases and controls were patients ≥18 years of age with central lines in situ for at least 48hrs from date of admission. Case definition was based on National Healthcare Safety Network (NHSN) framework to diagnose Bloodstream Infection (BSI) and CLABSI events. Controls had to be admitted within 30 days of the date of admission of the case patients and should not have developed CLABSI. Cases were matched to controls on a 1:2 ratio.

**Results:**

127 cases and 252 controls were included in the analysis. Cases and controls did not differ in age, gender, BMI, presence of diabetes mellitus or presently enforced infection prevention measures (e.g. Central line bundle care). More cases were receiving chemotherapy (10.2% versus 0.8%, p< 0.001), were on TPN (17.3% versus 8.3%, p=0.015) and had been admitted to critical care (73.2% versus 60.7%, p=0.017). Cases were also more likely to have peripherally inserted central venous catheters (37% versus 25%, p=0.017) and have the insertion done in the radiology department under radiological guidance (69.3% versus 55.2%, p=0.011). The median length of stay (LOS) was 44 days (IQR: 0 – 86.8) for cases and 19 days (IQR: 0 - 66.6) for controls (p< 0.001). Inpatient mortality was 25.2% (n=32) for cases 13.9% (n=35) for controls (p-value < 0.010). In multivariate analysis, receiving chemotherapy (OR 11.1, 95%CI: 2.2 – 54.3, p=0.003), being admitted to intensive care unit (ICU) (OR 2.0, 95%CI: 1.1 – 3.8, p=0.019), having a Peripherally Inserted Central Cather (OR 1.8, 95% CI 1.0-3.4, p=0.045), and being colonized with MRSA (OR 1.9, 95%CI: 1.2 – 3.2, p=0.013) were associated with healthcare associated CLABSI.

**Conclusion:**

Novel approaches are required to reduce risk of healthcare associated CLABSI, focusing on interventions for chemotherapy administration, care within ICUs and PICC lines.

**Disclosures:**

**All Authors**: No reported disclosures

